# Protective effects of astaxanthin on skin deterioration

**DOI:** 10.3164/jcbn.17-35

**Published:** 2017-06-20

**Authors:** Kumi Tominaga, Nobuko Hongo, Mayuko Fujishita, Yu Takahashi, Yuki Adachi

**Affiliations:** 1 AstaReal Co., Ltd., 55 Yoko-hoonji, Kamiichi-machi, Nakaniikawa-gun, Toyama 930-0397, Japan

**Keywords:** astaxanthin, inflammatory cytokines, wrinkle formation, skin elasticity, interleukin-1α

## Abstract

Astaxanthin is a carotenoid with potent antioxidant and anti-inflammatory activity. To evaluate the anti-inflammatory effect of astaxanthin on skin deterioration, we confirmed its role in epidermal-dermal interactions *in vitro*. Astaxanthin treatment suppressed ultraviolet B (UVB)-induced inflammatory cytokine secretion in keratinocytes, and matrix metalloproteinase-1 secretion by fibroblasts cultured in UVB-irradiated keratinocyte medium. To verify these findings, we conducted a 16-week clinical study with 65 healthy female participants. Participants were orally administered either a 6 mg or 12 mg dose of astaxanthin or a placebo. Wrinkle parameters and skin moisture content significantly worsened in the placebo group after 16 weeks. However, significant changes did not occur in the astaxanthin groups. Interleukin-1α levels in the stratum corneum significantly increased in the placebo and low-dose groups but not in the high-dose group between weeks 0 and 16. This study was performed in Japan from August to December, when changing environmental factors, such as UV and dryness, exacerbate skin deterioration. In conclusion, our study suggests that long-term prophylactic astaxanthin supplementation may inhibit age-related skin deterioration and maintain skin conditions associated with environmentally induced damage via its anti-inflammatory effect. (UMIN Clinical Trials Registry ID: UMIN000018550)

## Introduction

Astaxanthin is a naturally occurring xanthophyll carotenoid that was originally isolated from lobsters by Kuhn and Sorensen.^(1)^ It is found in crustaceans, such as shrimps and crabs, and fish, such as salmon and sea bream. The anti-inflammatory activity of astaxanthin, which is based on its antioxidant properties, has been implicated in improving lifestyle-related diseases and managing health. Furthermore, astaxanthin has anti-aging effects.^(2)^

Mechanisms of skin aging are classified into physiological and photoaging.^(3–5)^ Skin aging manifests as wrinkles, degradation of elasticity, and age spots (liver spots). Physiological skin aging occurs over the whole body and is a result of decreased cellular metabolism. Photoaging occurs in ultraviolet (UV)-irradiated areas of the skin, such as the face. It is primarily due to collagen and elastic fiber degradation that occurs because of matrix metalloproteinases (MMPs) secreted from dermal fibroblasts and epidermal keratinocytes in response to UV irradiation.^(6,7)^ In addition, MMP secretion and activation are stimulated by the various inflammatory cytokines secreted from keratinocytes by reactive oxygen species (ROS) from UV-irradiated cells.^(8)^ Skin inflammation is triggered by oxidative stress that results from stimuli such as UV exposure and skin dryness. Thus, suppression of inflammatory cytokines by oxidative stress inhibition is crucial for inhibiting age-related skin deterioration.

The effects of astaxanthin on hyper-pigmentation suppression, melanin synthesis and photoaging inhibition, and wrinkle formation reduction have been reported in several clinical studies.^(9,10)^ In this study, we conducted *in vitro* and *in vivo* studies to investigate the effects of astaxanthin on skin deterioration. We assessed the impact of astaxanthin on inflammatory cytokine and MMP-1 expression in UVB-irradiated human keratinocytes and human dermal fibroblasts, respectively. Furthermore, we conducted a randomized, double-blind, parallel-group, placebo-controlled study involving 65 healthy female participants for 16 weeks to investigate the *in vivo *effect of oral astaxanthin supplementation.

## Materials and Methods

### Chemicals and materials

Minimum essential medium alpha (MEMα) and Dulbecco’s modified Eagle’s medium (DMEM) were purchased from Life Technologies (Grand Island, NY), and fetal bovine serum (FBS) was obtained from Nichirei Biosciences (Tokyo, Japan). Human epidermal keratinocytes and KG2 medium were obtained from Kurabo (Osaka, Japan). Human dermal fibroblasts were obtained from the Riken BRC (Tsukuba, Japan). Dimethyl sulfoxide (DMSO) was purchased from Wako Pure Chemical Industries (Osaka, Japan). The enzyme-linked immunosorbent assay (ELISA) kits for interleukin (IL)-1α, IL-6, IL-8, and tumor necrosis factor (TNF)-α were purchased from R&D Systems (Minneapolis, MN). The MMP-1 ELISA kit was purchased from GE Healthcare (Buckinghamshire, England). Mammalian protein extraction reagent (M-PER) and the BCA protein assay kit were purchased from Thermo Scientific Pierce (Rockford, IL). Astaxanthin and all other reagents were purchased from Sigma-Aldrich (St. Louis, MO).

### Keratinocyte culture and UVB irradiation

 Keratinocytes were cultured in KG2 medium according to the manufacturer’s protocols and incubated at 37°C and 5% CO_2_. Cells were plated on 60-mm dishes at a density of 3.5 × 10^5^ cells/dish and incubated for 48 h before UVB treatment. Fresh KG2 medium containing various concentrations of astaxanthin (0, 1, 5 and 10 µM) and 0.5% (v/v) DMSO as a vehicle was then added to keratinocytes, and cells were incubated for another 4 h. Keratinocytes were washed once with phosphate-buffered saline (PBS), 500 µl PBS was added, and the cells were then irradiated with 5 mJ/cm^2^ UVB. PBS was immediately replaced with fresh KG2 medium containing various concentrations of astaxanthin (0, 1, 5 and 10 µM) and 0.5% DMSO, and keratinocytes were cultured for 24 h. The culture medium was harvested and frozen at –20°C until subsequent analysis. Keratinocytes were rinsed once with PBS and lysed in 1.0 ml M-PER.

### UVB source

Keratinocyte irradiation was performed using two UVB lamps (TL20W/12RS; Philips Lighting, Holding, Amsterdam, Netherlands). UVB irradiance was measured using the UV light meter UV-340 (Lutron Electronics, Coopersburg, PA).

### Indirect treatment of fibroblasts with UVB-irradiated keratinocytes

Fibroblasts were cultured in MEMα supplemented with 10% FBS at 37°C and 5% CO_2_. Cells were grown in 48-well plates at a density of 2.4 × 10^3^ cells/well and cultured overnight before treatment. To investigate the indirect effect of astaxanthin on MMP-1 production by fibroblasts as mediated by keratinocytes, keratinocytes were pre-treated with astaxanthin and irradiated with UVB, as described above. Following UVB irradiation, keratinocytes were cultured for 24 h in fresh DMEM containing various concentrations of astaxanthin and 0.5% DMSO. The keratinocyte culture medium was collected and immediately transferred to fibroblasts. After 48 h, the fibroblast culture medium was harvested and frozen at –20°C until the MMP-1 assay. Fibroblasts were rinsed once with PBS and lysed in 1.0 ml M-PER.

### ELISA and protein assay

Levels of IL-1α, IL-6, IL-8 and TNF-α secreted from keratinocytes were measured by ELISA as per the manufacturer’s instructions. MMP-1 levels secreted from fibroblasts were also measured by ELISA according to the manufacturer’s instructions. ELISA data were normalized to the total protein content in the cell lysate corresponding to each sample medium. Protein concentrations were measured using the BCA protein assay kit.

### Clinical study of astaxanthin supplementation

A randomized, double-blind, parallel-group, placebo-controlled study was conducted to evaluate the effects of astaxanthin on wrinkle formation and other aspects of skin damage and aging. Before starting the clinical test, the wrinkle grades on the lower and outer angle eyelids of subject candidates were evaluated by a trained expert. Sixty-five healthy female participants (age, 35–60 years) with a wrinkle grade of 2.5 to 5.0 were enrolled. Written informed consent was obtained, and the study was approved by the Institutional Review Board of the Tokyo Synergy Clinic, Tokyo, Japan.

Measurements were performed at three key points in the study: before treatment (week 0), after 8 weeks (week 8), and after 16 weeks (week 16). A biochemical examination of blood serum chemistry was also performed to assess astaxanthin supplementation safety at weeks 0 and 16. The study period was from August to December 2015 at the research institution in Osaka, Japan.

### Material for oral supplementation

The material for oral supplementation contained AstaReal^®^ Oil 50F (Fuji Chemical Industries, Toyama, Japan), 5% w/w astaxanthin *Haematococcus pluvialis* extract, and canola oil as soft gel capsules. Participants were randomly assigned to one of three groups: high-dose (*n* = 22), low-dose (*n* = 22), or placebo (*n* = 21), in which participants were administered daily oral supplements containing 12, 6 or 0 mg astaxanthin, respectively, for 16 weeks.

### Wrinkle analysis and instrumental measurements

 For screening of subjects for the wrinkle grade, evaluators used the wrinkle grade standard proposed by the Japanese Cosmetic Science Society^(11)^ using a photo of each participant that was taken using VISIA^®^ Evolution (Canfield Scientific, NJ). During the study period, the following measurements were performed: replicas of wrinkles of the same area were obtained, and four parameters (area ratio of all wrinkles, mean depth of the deepest wrinkle, maximum depth of the deepest wrinkle, and mean depth of all wrinkles) were calculated by image analysis using PRIMOS LITE (GFMesstechnik, Teltow, Germany). Capacitance of the cheek, which indicates skin moisture content, was measured using a skin hygrometer SKICON-200EX (YAYOI, Tokyo, Japan). Transepidermal water loss at the cheek was measured using AS-CT1 (Asahibiomed, Tokyo, Japan). Skin elasticity was measured using a Cutometer MPA560 (Courage+Khazaka electronic, Cologne, Germany), and three parameters, R2, R6 and R7, which represent gross elasticity, portion of the viscoelasticity, and biological elasticity, respectively, were calculated. The stratum corneum of each subject was obtained using the adhesive Skin Checker (Promotool Corporation, Tokyo, Japan), and IL-1α levels in the stratum corneum were measured by ELISA according to the manufacturer’s instructions.

### Statistical analyses

All data are reported as mean ± SD. Statistical tests were performed using Dunnett’s test or Student’s *t* test, and a *p* value of <0.05 was considered to be statistically significant.

## Results

### Effects of astaxanthin supplementation on inflammatory cytokine production in UVB-irradiated keratinocytes

**Fig. 1:**
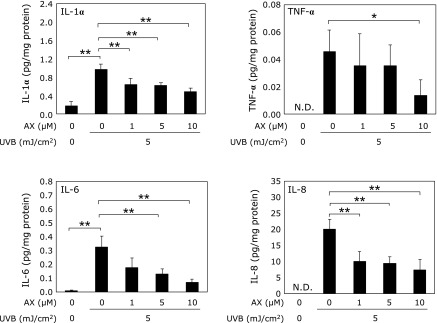
Effect of astaxanthin on cytokine production in UVB-irradiated keratinocytes. Each value represents mean value ± SD, **p<0.01 and *p<0.05 by ANOVA/Dunnett’s test. ANOVA, analysis of variance; AX, astaxanthin; IL, interleukin; TNF, tumor necrosis factor; UV, ultraviolet.

Keratinocytes were treated for 4 h with 0, 1, 5 or 10 µM astaxanthin and then irradiated with 5 mJ/cm^2^ UVB. The culture medium was collected, and the levels of inflammatory cytokines, including IL-1α, IL-6, IL-8 and TNF-α, were measured. All cytokine levels in the culture medium significantly increased following UVB irradiation compared with those in the non-irradiated medium. IL-1α, IL-6, IL-8 and TNF-α levels significantly decreased following treatment with astaxanthin in a dose-dependent manner (Fig. 1).

### Effects of astaxanthin supplementation on fibroblast MMP-1 production

**Fig. 2:**
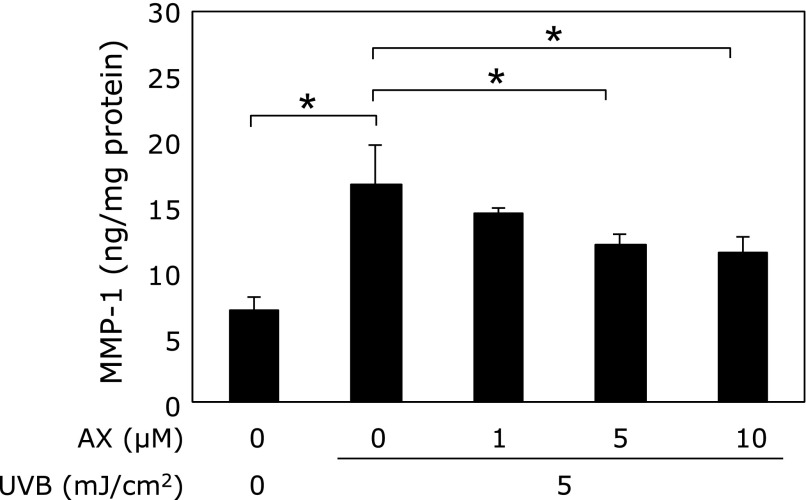
Effect of conditioned media from UVB-irradiated keratinocytes treated with or without astaxanthin on MMP-1 production in cultured fibroblasts. Each value represents mean value ± SD, *p<0.01 by ANOVA/Dunnett’s test. ANOVA, analysis of variance; AX, astaxanthin; MMP, matrix metalloproteinase; UV, ultraviolet.

Fibroblasts were incubated with media derived from keratinocytes that were treated with 0, 1, 5 or 10 µM astaxanthin before and after irradiation with UVB. The fibroblast culture medium was then collected and assessed for MMP-1 production. MMP-1 levels significantly increased in the medium derived from UVB-irradiated keratinocytes. However, MMP-1 levels significantly decreased in a dose-dependent manner in the presence of medium derived from astaxanthin-treated keratinocytes (Fig. 2).

### Clinical efficacy of oral astaxanthin supplementation on facial skin

**Fig. 3:**
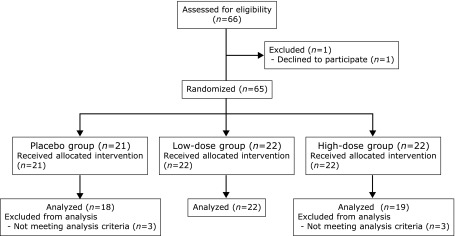
Flow diagram of the clinical trial.

**Fig. 4:**
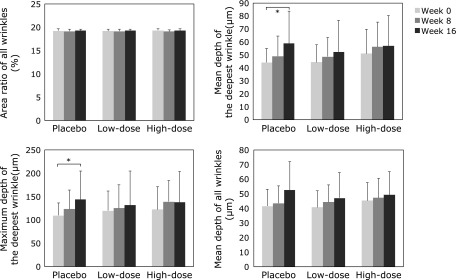
Effect of oral astaxanthin supplementation on wrinkle parameters from replica image analysis. Each value represents mean value ± SD, *p<0.05 by ANOVA/Dunnett’s test. ANOVA, analysis of variance.

**Fig. 5:**
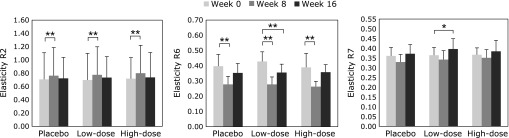
Effect of oral astaxanthin supplementation on skin elasticity. Each value represents mean value ± SD, **p<0.01 and *p<0.05 by ANOVA/Dunnett’s test. ANOVA, analysis of variance.

**Fig. 6:**
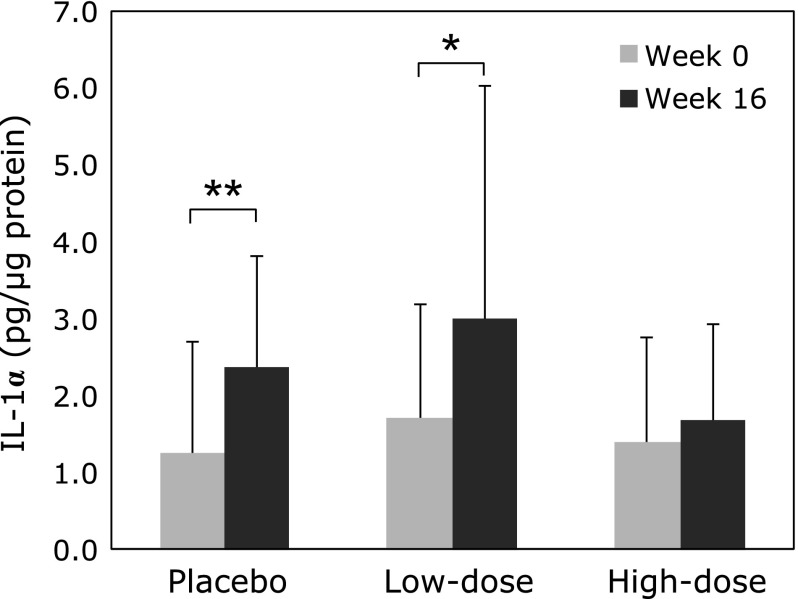
Effect of oral astaxanthin supplementation on IL-1α in the stratum corneum measured by ELISA. Each value represents mean value ± SD, **p<0.01 and *p<0.05 by paired *t* test. ELISA, enzyme-linked immunosorbent assay; IL, interleukin.

Of 66 individuals who volunteered for the study, 59 participants were included in our clinical study (Fig. 3). The wrinkle grades were 3.45 ± 0.71 in the placebo group, 3.43 ± 0.70 in the low-dose group, and 3.38 ± 0.68 in the high-dose group at week 0. Mean and maximum depths of the deepest wrinkle significantly deteriorated in the placebo group at week 16 compared with those at week 0 (Fig. 4). Furthermore, skin moisture content displayed significant deterioration in the placebo group at week 16 (204.9 ± 54.2 µS) compared with that at week 0 (264.7 ± 100.3 µS). In contrast, significant deteriorations in wrinkle parameters from replica image analysis and skin moisture content (data not shown) were not observed in the astaxanthin-treated groups, indicating that astaxanthin maintained skin conditions during the study period. Skin elasticity was evaluated using three parameters, R2, R6 and R7. R2 and R6 values significantly improved in all groups at week 8, but R6 and R7 values significantly improved at week 16 compared with those at week 0 only in the low-dose group (Fig. 5). In addition, IL-1α levels in the stratum corneum significantly deteriorated in the placebo and low-dose groups at week 16, but did not change in the high-dose group during the study period (Fig. 6). There were no changes in transepidermal water loss in any group (data not shown). No measured parameters, including wrinkle parameters, skin moisture content, transepidermal water loss, skin elasticity, and IL-1α levels, displayed significant differences when comparing among the groups at weeks 0, 8 and 16.

### Stratified analyses

**Fig. 7:**
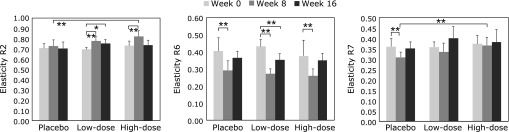
Stratified analysis of skin elasticity parameters of the cheek. Each value represents mean value ± SD, **p<0.01 and *p<0.05 by ANOVA/Dunnett’s test. R2, R6 and R7 represent gross elasticity, portion of the visco-elasticity, and biological elasticity, respectively. ANOVA, analysis of variance.

The stratified analysis was conducted for participants whose capacitance at week 0 was higher than the mean capacitance of all subjects (264.8 µS). There were 10 participants in the placebo group, 7 in the low-dose group and 11 in the high-dose group. Mean depth of the deepest wrinkle, maximum depth of the deepest wrinkle and mean depth of all wrinkles significantly worsened in the placebo group at week 16 (68.6 ± 23.0, 172.0 ± 53.6 and 59.9 ± 19.8 µm, respectively) compared with those at week 0 (43.6 ± 11.6, 108.6 ± 29.2 and 42.9 ± 13.7 µm, respectively). In contrast, significant worsening of wrinkle parameters was not observed in the astaxanthin-treated group (data not shown). R2 and R6 values significantly improved only in the low-dose group at week 16, and the R7 value deteriorated only in the placebo group at week 8 compared with those at week 0 (Fig. 7). Moreover, the R2 value displayed a significant improvement in the high-dose group (*p* = 0.001) at week 8 compared with that in the placebo group. In addition, the R7 value displayed a significant improvement in the high-dose group at week 8 (*p* = 0.002) and a tendency to improve in the low-dose group at week 16 (*p* = 0.100) compared with that in the placebo group.

### Safety evaluation of astaxanthin supplementation

Results of the biochemical examination of blood are summarized in Table 1. There were no abnormal changes in blood parameters observed during the study period, and no serious adverse events were reported.

## Discussion

We conducted *in vitro* and *in vivo* studies to investigate the effects of astaxanthin, a natural antioxidant, on skin deterioration observed in aged skin. We demonstrated that pre- and post-treatment with astaxanthin dose-dependently suppressed the secretion of inflammatory cytokines from UVB-irradiated keratinocytes. Furthermore, MMP-1 production by fibroblasts that were treated with medium from UVB-irradiated keratinocytes with astaxanthin treatment decreased in a dose-dependent manner.

Inflammatory cytokines released from epidermal keratinocytes stimulate dermal fibroblasts and keratinocytes in an autocrine manner and then upregulate messenger ribonucleic acid expression, protein, and enzymatic activity levels of MMPs, such as MMP-1, MMP-3 and MMP-9. MMPs subsequently affect collagen and elastic fibers, leading to the formation of wrinkles.^(12)^ UV-induced wrinkle formation is markedly inhibited by elastase activity suppression in degrading elastic fibers. Previous study indicated that qualitative and quantitative changes in elastic fibers caused wrinkle formation. Our *in vitro* study indicated that UVB irradiation-induced inflammation and inflammatory cytokine-stimulated MMP-1 levels were suppressed by astaxanthin. These results corroborated those of previous studies.^(13–16)^ In addition, it was reported that long-term treatment with astaxanthin prevented the accumulation of age-related oxidative stress and inflammatory response in aging mice.^(17)^ Thus, based on these collective results, oral astaxanthin supplements are expected to inhibit inflammation-mediated skin deterioration, such as wrinkle formation and skin moisture decline, that appears in aged skin.

Next, we conducted a randomized, double-blind, parallel-group, placebo-controlled study with 65 healthy female subjects for 16 weeks to verify the effects of oral astaxanthin supplementation on skin integrity. We determined that skin moisture content and deep wrinkles were not significantly changed in the astaxanthin-supplemented groups, whereas these parameters significantly worsened in the placebo group during the study period. Furthermore, IL-1α levels in the stratum corneum were maintained only in the high-dose group. In addition, skin elasticity improvements were observed in high-dose group compared with that of the placebo group in participants with high skin moisture content.

It is well known that both UV radiation and dryness cause progression of wrinkle formation. UV irradiation contributes to wrinkle formation by inducing MMP secretion from dermal fibroblasts via cytokines, such as IL-1α, IL-6, and TNF-α, released by UVB-exposed keratinocytes.^(4)^ In the region where this study was conducted, daytime UV light is the strongest between May and September, and the air humidity declines from August, reaching the lowest levels between December and April. Our clinical trial was conducted from August to December, a period during which exposure to strong UV radiation during the summer is followed by a decrease in humidity during the autumn and winter months. As a result of these changing environmental factors, skin barrier function declines and skin dryness progresses.^(18)^ Exposure to low humidity induces epidermal IL-1α synthesis, and water flux in the epidermis might be the first signal to induce IL-1α synthesis in the epidermis.^(19)^ IL-1α may also induce other proinflammatory cytokines, such as IL-6 and IL-8.^(20)^ Thus, the IL-1α level in the stratum corneum is linked to skin dryness, which we also observed in the placebo and low-dose groups, even though wrinkle parameters in the astaxanthin-treatment groups were not significantly altered.

With respect to safety, no adverse events were observed with oral astaxanthin supplementation of 12 mg/day for 16 weeks. Our results confirm the long-term safety of astaxanthin as an oral supplement.

In conclusion, our findings indicated that astaxanthin inhibited inflammatory cytokine secretion from epidermal keratinocytes and MMP-1 secretion by dermal fibroblasts in response to UVB irradiation. These mechanisms underlie clinical study results that demonstrated that the suppression of seasonal deterioration of wrinkles and skin moisture and the improvements in skin elasticity were accompanied by sustaining low IL-1α levels in the epidermal corneum. Thus, long-term astaxanthin supplementation may prophylactically inhibit skin deterioration induced over time by environmental damage and consequently retard the skin aging process via its anti-inflammatory effect.

## Figures and Tables

**Table 1:** Summary of the biochemical blood exam

	Group	*n*	Measured value		Comparison between week 0 and 16	Intergroup comparison of change amount
			Week 0	Week 16		
			Mean SD	Mean SD	*p* value	*p* value
Total bilirubin (mg/dl)	Placebo	18	0.76 ± 0.24	0.67 ± 0.15	0.064	
	Low-dose	22	0.75 ± 0.22	0.74 ± 0.21	0.696	0.370
	High-dose	19	0.71 ± 0.20	0.71 ± 0.16	1.000	0.791

AST (U/L)	Placebo	18	19.89 ± 3.48	21.56 ± 5.19	0.053	
	Low-dose	22	20.82 ± 7.22	21.32 ± 4.22	0.678	0.979
	High-dose	19	18.21 ± 4.81	20.00 ± 3.80	0.008*	0.457

ALT (U/L)	Placebo	18	14.11 ± 4.54	15.78 ± 7.95	0.219	
	Low-dose	22	17.64 ± 11.56	15.77 ± 7.12	0.247	1.000
	High-dose	19	13.95 ± 4.96	14.79 ± 4.22	0.353	0.860

ALP (U/L)	Placebo	18	185.28 ± 60.11	191.06 ± 59.17	0.234	
	Low-dose	22	191.68 ± 71.31	185.32 ± 59.43	0.325	0.923
	High-dose	19	179.58 ± 38.65	193.26 ± 43.91	0.001*	0.989

LD (LDH) (U/L)	Placebo	18	166.83 ± 16.04	161.72 ± 20.36	0.149	
	Low-dose	22	181.82 ± 22.56	173.95 ± 25.23	0.016*	0.291
	High-dose	19	165.00 ± 31.11	166.47 ± 36.13	0.777	0.823

γ-GT (U/L)	Placebo	18	24.17 ± 24.06	23.22 ± 21.29	0.365	
	Low-dose	22	27.09 ± 27.56	22.41 ± 15.60	0.151	0.979
	High-dose	19	16.00 ± 3.54	15.53 ± 4.05	0.283	0.224

CK (U/L)	Placebo	18	92.11 ± 46.07	96.39 ± 58.94	0.438	
	Low-dose	22	92.27 ± 44.62	100.86 ± 46.12	0.318	0.933
	High-dose	19	96.16 ± 39.00	89.53 ± 29.70	0.275	0.861

Total protein (g/dl)	Placebo	18	7.40 ± 0.38	7.50 ± 0.41	0.252	
	Low-dose	22	7.50 ± 0.32	7.57 ± 0.38	0.296	0.774
	High-dose	19	7.39 ± 0.43	7.66 ± 0.36	0.012*	0.356

A/G	Placebo	18	1.45 ± 0.18	1.41 ± 0.20	0.304	
	Low-dose	22	1.41 ± 0.11	1.43 ± 0.18	0.718	0.967
	High-dose	19	1.43 ± 0.19	1.41 ± 0.21	0.453	0.997

ALB (g/dl)	Placebo	18	4.36 ± 0.22	4.37 ± 0.20	0.895	
	Low-dose	22	4.39 ± 0.21	4.43 ± 0.18	0.346	0.467
	High-dose	19	4.33 ± 0.21	4.45 ± 0.21	0.010*	0.305

Creatinine (mg/dl)	Placebo	18	0.69 ± 0.10	0.64 ± 0.08	0.001*	
	Low-dose	22	0.67 ± 0.08	0.64 ± 0.07	0.011*	0.999
	High-dose	19	0.69 ± 0.08	0.65 ± 0.10	0.003*	0.995

Urea nitrogen (mg/dl)	Placebo	18	13.28 ± 3.01	12.56 ± 2.66	0.200	
	Low-dose	22	13.27 ± 2.39	12.73 ± 2.90	0.352	0.970
	High-dose	19	13.00 ± 2.60	12.11 ± 2.42	0.145	0.826

Uric acid (mg/dl)	Placebo	18	4.36 ± 1.05	4.22 ± 1.11	0.269	
	Low-dose	22	4.60 ± 0.73	4.45 ± 0.92	0.071	0.701
	High-dose	19	4.23 ± 0.92	4.28 ± 0.97	0.506	0.974

Fasting glucose (mg/dl)	Placebo	18	88.06 ± 7.69	87.89 ± 9.50	0.886	
	Low-dose	22	87.18 ± 8.54	87.23 ± 9.73	0.975	0.967
	High-dose	19	87.26 ± 7.27	90.05 ± 10.38	0.206	0.726

HbA1c (%)	Placebo	18	5.37 ± 0.28	5.37 ± 0.32	1.000	
	Low-dose	22	5.34 ± 0.20	5.32 ± 0.25	0.411	0.794
	High-dose	19	5.31 ± 0.23	5.27 ± 0.25	0.187	0.474

Total cholesterol (mg/dl)	Placebo	18	234.22 ± 50.79	231.50 ± 49.64	0.286	
	Low-dose	22	230.41 ± 32.68	228.82 ± 28.91	0.590	0.960
	High-dose	19	211.37 ± 24.99	218.53 ± 28.03	0.059	0.446

Triglyceride (mg/dl)	Placebo	18	76.44 ± 27.65	72.56 ± 30.31	0.428	
	Low-dose	22	71.00 ± 24.80	71.14 ± 30.84	0.977	0.983
	High-dose	19	68.84 ± 24.26	64.26 ± 28.39	0.399	0.606

HDL cholesterol (mg/dl)	Placebo	18	72.78 ± 11.40	76.22 ± 13.32	0.011*	
	Low-dose	22	75.23 ± 10.77	80.41 ± 12.40	0.008*	0.540
	High-dose	19	73.63 ± 15.84	83.00 ± 16.21	0.000*	0.247

LDL cholesterol (mg/dl)	Placebo	18	140.89 ± 47.68	141.11 ± 47.39	0.939	
	Low-dose	22	136.77 ± 30.67	136.32 ± 30.56	0.830	0.872
	High-dose	19	119.79 ± 21.30	122.53 ± 24.68	0.419	0.193

Arteriosclerosis index	Placebo	18	2.00 ± 0.76	1.93 ± 0.80	0.159	
	Low-dose	22	1.87 ± 0.54	1.76 ± 0.57	0.059	0.642
	High-dose	19	1.73 ± 0.62	1.55 ± 0.58	0.005*	0.144

Na (mEq/L)	Placebo	18	140.61 ± 1.33	139.17 ± 1.72	0.000*	
	Low-dose	22	140.32 ± 1.62	140.55 ± 1.26	0.424	0.019*
	High-dose	19	140.11 ± 1.59	140.00 ± 1.91	0.772	0.217

K (mEq/L)	Placebo	18	4.12 ± 0.26	4.18 ± 0.27	0.373	
	Low-dose	22	4.24 ± 0.32	4.32 ± 0.27	0.178	0.222
	High-dose	19	4.17 ± 0.19	4.26 ± 0.32	0.258	0.567

Cl (mEq/L)	Placebo	18	102.39 ± 1.72	102.28 ± 1.74	0.742	
	Low-dose	22	102.86 ± 1.55	103.23 ± 1.85	0.257	0.198
	High-dose	19	102.53 ± 1.58	102.32 ± 2.00	0.600	0.997

Ca (mEq/L)	Placebo	18	9.58 ± 0.28	9.39 ± 0.23	0.004*	
	Low-dose	22	9.74 ± 0.35	9.50 ± 0.26	0.000*	0.392
	High-dose	19	9.67 ± 0.38	9.62 ± 0.34	0.434	0.034*

Serum iron (µg/dl)	Placebo	18	122.61 ± 45.95	94.22 ± 35.22	0.009*	
	Low-dose	22	106.05 ± 40.02	118.32 ± 46.81	0.226	0.126
	High-dose	19	105.11 ± 39.92	102.58 ± 39.65	0.752	0.760

*p<0.05 by student’s *t* test or ANOVA/Dunnett’s test. AST, aspartate aminotransferase; ALT, alanine aminotransferase; ALP, alkaline phosphatase; LD, lactate dehydrogenase; γ-GT, gumma-glutamyl transferase; CK, creatine kinase; A/G, albumin/globulin ratio; ALB, albumin; HbA1c, hemoglobin A1c; HDL, high density lipoprotein; LDL, low density lipoprotein; Na, sodium ; K, potassium; Cl, chlorine; Ca, calcium; ANOVA, analysis of variance.

## Abbreviations

BCA: bicinchoninic acid
DMEM: Dulbecco’s modified Eagle’s medium
DMSO: dimethyl sulfoxide
ELISA: enzyme-linked immunosorbent assay
FBS: fetal bovine serum
IL: interleukin
MEMα: minimum essential medium alpha
MMP: matrix metalloproteinase
M-PER: mammalian protein extraction reagent
PBS: phosphate-buffered saline
ROS: reactive oxygen species
TNF: tumor necrosis factor
UV: ultraviolet
